# Severe Epstein–Barr virus encephalitis with peripheral nerve damage: A case report

**DOI:** 10.1097/MD.0000000000040804

**Published:** 2024-12-13

**Authors:** Aidi Luo, Chang Li, Jing Zhao, Yujuan Wu, Rong Fu

**Affiliations:** a Department of Neurology, The Second People’s Hospital of Guiyang (Jinyang Hospital), Guiyang, Guizhou, China.

**Keywords:** Epstein–Barr virus, peripheral nerve, viral encephalitis

## Abstract

**Rationale:**

Epstein–Barr virus (EBV) is a B-lymphotropic double-stranded DNA virus. Most people infected with EBV are asymptomatic infection. Its clinical symptoms are rarely manifested as EBV encephalitis, and peripheral nerve damage is even rarer.

**Patient concerns:**

We report a case of a 49-year-old woman with a history of fever. The initial symptoms were numbness and weakness in the right hand, followed by slurred speech. Electromyography showed severe damage to the median nerve of the right wrist (involving sensory and motor fibers). Magnetic resonance imaging revealed multiple lesions in the bilateral cerebral hemispheres on T2 FLAIR images, and T1-enhanced images showed abnormal enhancement of the adjacent leptomeninges. Human herpesvirus 4 (EBV) has been detected in the cerebrospinal fluid using metagenomic next-generation sequencing (NGS). After antiviral treatment, the patient’s symptoms continued to worsen.

**Diagnosis:**

Severe EBV encephalitis complicated with peripheral nerve damage.

**Interventions:**

Antiviral, hormone therapy.

**Outcomes:**

At the patient’s condition progressed, a new infarction occurred in the right frontal lobe lesion, with repeated high fever, rapid deterioration of multiple organ function, sudden respiratory failure, cardiac arrest, and death.

**Lessons:**

In patients with signs of encephalitis, cerebrospinal fluid NGS should be used as early as possible to confirm the diagnosis of EBV encephalitis. Timely and accurate treatment of central nervous system infections is expected to reduce the mortality rate and improve the quality of life of patients in later stages.

## 1. Introduction

Epstein–Barr virus (EBV) is a common human type 4 herpes virus, and approximately 90% of the population has asymptomatic infections, but it increases the risk of certain cancers and autoimmune diseases.^[1]^ 0.5% to 7.5% of patients have nervous system involvement, which can directly damage the brain, meninges, spinal cord, cranial nerves, and peripheral nerves. At present, EBV encephalitis cases are mostly reported in children and in some immunocompromised adults. The clinical symptoms of EBV encephalitis are nonspecific, and the severity of the disease varies. Mild cases are self-limiting, and severe cases can lead to sequelae or death. This report describes a rare case of severe EBV encephalitis with peripheral nerve damage.

## 2. Case presentation

**Figure 1.**
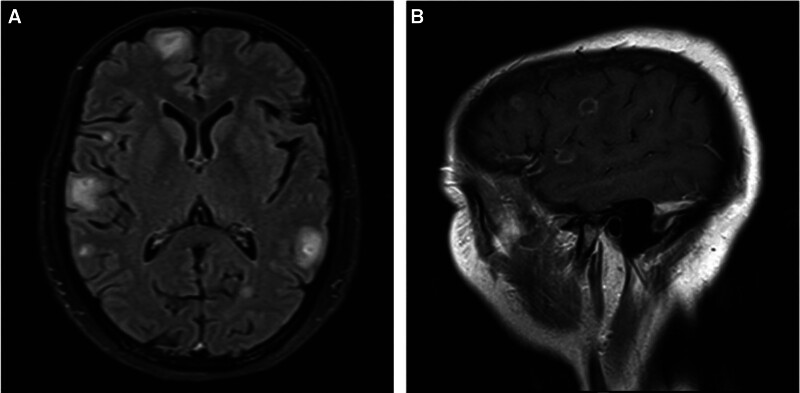
(A) Brain magnetic resonance T2 FLAIR sequence showed multiple lesions in bilateral cerebral hemispheres with high signal, including frontal lobe, temporal lobe, parietal lobe and occipital lobe. (B) T1-enhanced images showed abnormal enhancement of pia mater. T2 FLAIR = T2 fluid-attenuated inversion recovery.

A 49-year-old female presented with a fever of up to 38 °C 1 week ago. After 2 days of fever, she developed weakness in the right hand, inflexible activity, and numbness of the 3 fingers on the radial side of the right hand, which was not taken seriously. Two days before admission, the patient developed right hand weakness, the right hand could not hold the fist and grasp the items, and there was unclear speech. Physical examination was performed after admission in September 2024. The body temperature was 36.8 °C, heart rate was 90 beats/min, breathing rate was 18 beats/min, and blood pressure was 128/84 mmHg (1 mmHg = 0.133 kPa). Cardiopulmonary abdominal examination revealed no abnormalities, and the superficial lymph nodes did not show swelling. Physical examination of the nervous system showed that the speech was unclear; the right thumb, index finger, and middle finger could not be flexed; the thumb could not be opposed and abducted; the pain of the right thumb, index finger, middle finger, and thenar decreased; and the pathological signs were negative. C-reactive protein increased (8.30 mg/L, normal range 0–6 mg/L), complement C4 increased (0.44 g/L, normal range 0.1–0.4 g/L), immunoglobulin A increased (5.09 g/L, normal range 0.7–4 g/L). Lumbar puncture pressure 170 mmH_2_O, cerebrospinal fluid routine: total cell count 24 × 10^6^/L, nucleated cell count 6 × 10^6^/L. Cerebrospinal fluid biochemistry (protein was 0.38 g/L, normal range 0.15–0.45 g/L), TORCH virus, general bacterial smear, ink staining, acid-fast staining, and bacterial culture were not abnormal. Cerebrospinal fluid next-generation sequencing (NGS) display: human herpes virus type 4 (EBV), with 16,503 sequences. Autoimmune encephalitis antibody, MOG antibody and OCB tests were negative. There were no obvious abnormalities in blood routine, liver and kidney function, electrolytes, myocardial enzymes, blood glucose, blood lipids, coagulation analysis, ANA, ANCA, 4 items of rheumatism, thyroid function and antibodies, tumor markers, paraneoplastic syndrome-related antibodies, 5 items of vasculitis, anticardiolipin antibodies, 8 items of infectious diseases, stool and urine routine. Craniocerebral magnetic resonance imaging (MRI) showed multiple lesions in bilateral cerebral hemispheres (Fig. 1A), bilateral ethmoid sinus, right maxillary sinusitis, and abnormal enhancement of pia mater (Fig. 1B). Electromyography showed severe damage to the median nerve of the right wrist (involving sensory and motor fibers). EEG was normal. There were no obvious abnormalities on chest and abdomen CT or cervical and thoracic magnetic MRI.

**Figure 2.**
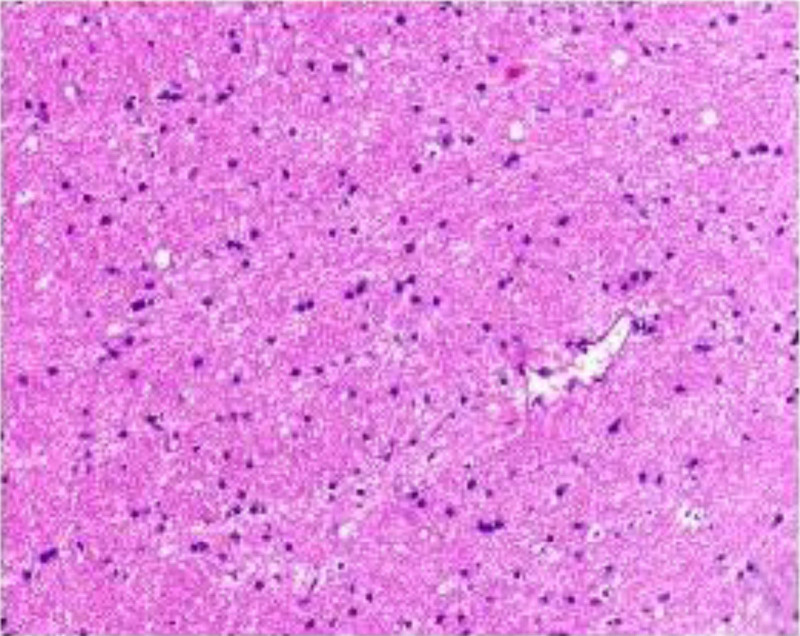
Pathology of brain tissue biopsy in patients: glial cell proliferation, focal calcification, a little lymphocyte infiltration near the small blood vessels, red blood cells and neutrophils at the edge.

**Figure 3.**
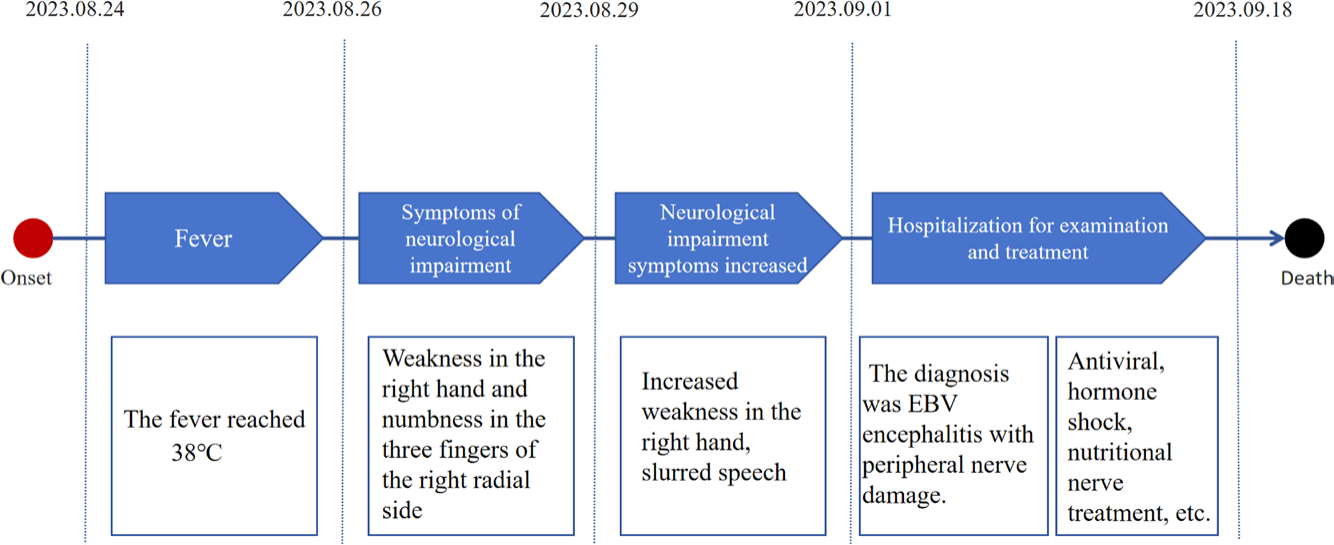
The timeline of medical history.

Based on the patient’s symptoms, signs, and related examination results, the diagnosis was EBV encephalitis with peripheral nerve damage. We used acyclovir 0.5 g (3 times/d) for antiviral therapy, methylprednisolone sodium succinate 1000 mg (1 time/d, continuous infusion for 5 days, reduced by half every 3 days), and vitamins B1and B12 to nourish the nerves. However, the patient’s symptoms did not improve significantly. MRI showed that some intracranial lesions were larger than before, and the right frontal lobe lesions showed infarction. An intracranial lesion biopsy was performed to exclude tumor lesions (Fig. 2), and the antiviral regimen was changed to ganciclovir 0.25 g (2 times/d). After 2 weeks of treatment, the symptoms of the patients did not significantly improve, and there was repeated high fever, rapid deterioration of multiple organ function, sudden respiratory failure, cardiac arrest, and death. Figure 3 shows the timeline of medical history.

## 3. Conclusions

Compared to central nervous system infections caused by enterovirus, herpes simplex virus, and Coxsackie virus, EBV-induced infections are relatively rare. EBV infection of the central nervous system can manifest as an acute or chronic course. Acute onset is due to the virus more direct invasion of brain, meninges, and spinal cord, multiple neural axon of the peripheral nerve, the most common disease is infectious mononucleosis; The chronic course of EBV infection is caused by the deposition of antigen-antibody immune complexes after EBV infection, secondary inflammatory reaction leading to demyelinating changes in brain tissue, or reactivation of the virus when the virus is latent in B lymphocytes and immunosuppression. It is most common in adults with a chronic active EBV infection.^[1,2]^ Severe cases not only affect the patient ‘s nerve tissue, but also lead to immune system involvement, leading to Guillain–Barre syndrome (GBS), multiple sclerosis, acute disseminated encephalomyelitis (ADEM), etc.^[3–6]^ EBV encephalitis accounts for 5% to 18% of acute viral encephalitis. They are often treated for mental symptoms. The course of the disease is mostly self-limiting, and imaging is nonspecific. Abu-Kasim et al reported that approximately 60% of patients with EBV-associated meningoencephalitis have abnormal CT or MRI findings, usually in the cerebral hemisphere, cerebellum, basal ganglia, but mortality is highest when the brain stem is involved.^[7]^ At present, clinical detection of EBV nucleic acids in cerebrospinal fluid or brain tissue is a common standard for clarifying EBV infection in the central nervous system. Metagenomic NGS, a new molecular diagnostic technique, is being increasingly used in clinical practice.

The patient had acute onset, with fever, speech disorder and peripheral nerve dysfunction as the main manifestations. Cranial MRI suggested extensive cortical and subcortical tissue involvement, excluding tumors, other pathogenic infections, autoimmune encephalitis, and other conditions. NGS revealed that the cerebrospinal fluid was positive for EBV DNA, and EBV encephalitis was considered to be associated with peripheral nerve damage. This effect was poor after antiviral and hormone-shock therapy. Our patient had no history of frequent or opportunistic infections, organ transplantation, no immunosuppressive drugs, or negative HIV test results. This report describes adult EBV encephalitis with normal immunity. The pathological basis of EBV encephalitis is inflammatory swelling and vascular inflammatory changes in the brain parenchyma, or is accompanied by immune demyelination. Viral infection-induced cytotoxic CD8 + lymphocyte infiltration into the pia mater or nerve tissue and deposition of antigen-antibody complexes may lead to immune damage. EBV-induced single peripheral nerve injury is rare, and patients show only median nerve damage. It is considered that immune-mediated peripheral nerve demyelination and axonal damage may occur following viral infection. Multiple reports have shown that EBV can induce GBS due to the immune response against peripheral nerve myelin or axons caused by precursor infection. Myelin or axons cross-react with peripheral nerve components in molecular simulations.^[5,8]^ In this report, the patient developed a new acute cerebral infarction, which may be related to EBV-induced vasculitis. However, antiviral and hormone-shock therapy cannot prevent the rapid progression of the disease. In 2017, Kano reported a case of central nervous system vasculitis associated with EBV infection, which was pathologically confirmed. HE staining showed perivascular lymphocyte infiltration, vascular wall thickening, and fibrinoid necrosis in the vascular wall.^[9]^ Ridha et al reported a case of acute cerebral infarction associated with EBV infection in a patient who received immunosuppressive agents after organ transplantation and died of recurrent infarction after antiviral treatment.^[10]^ Previous autopsy pathology found that monocyte infiltration around blood vessels and necrosis caused by immune response induced by EBV infection may be the main cause of death in a very small number of patients.

Currently, there is no treatment guideline for EBV encephalitis, and antiviral and symptomatic supportive treatment are the main clinical treatments. However, some studies have shown that antiviral therapy has no significant effect on the course of the disease and prognosis, and the antiviral efficacy of acyclovir and ganciclovir is controversial. The majority of EBV encephalitis patients with normal immune function have a good prognosis, with a mortality rate of approximately 10 %, but the results in immunocompromised patients are unknown.^[11]^ We suggest that in patients with signs of encephalitis, cerebrospinal fluid NGS tests should be used as soon as possible to confirm the diagnosis of EBV encephalitis. The immediate use of steroids and immunoglobulins as soon as possible may be beneficial for patients with life-threatening EBV encephalitis.^[12]^ For the treatment of chronic active EBV with high mortality and disability rate, hematopoietic stem cell transplantation may be an emerging strategy.^[13]^

## Acknowledgments

Science and Technology Fund of Guizhou Provincial Health Commission (Grant No. gzwkj2024-300), Graduate Workstation of Neurology, Zunyi Medical College (No. GZZ2017004).

## Author contributions

**Conceptualization:** Jing Zhao, Yujuan Wu.

**Data curation:** Chang Li.

**Formal analysis:** Yujuan Wu.

**Funding acquisition:** Rong Fu.

**Supervision:** Chang Li, Rong Fu.

**Writing – original draft:** Aidi Luo.

**Writing – review & editing:** Aidi Luo.
